# Engineering Nanoparticles to Reprogram the Tumor Immune Microenvironment for Improved Cancer Immunotherapy

**DOI:** 10.7150/thno.37568

**Published:** 2019-10-17

**Authors:** Madiha Saeed, Jing Gao, Yang Shi, Twan Lammers, Haijun Yu

**Affiliations:** 1 State Key Laboratory of Drug Research & Center of Pharmaceutics, Shanghai Institute of Materia Medica, Chinese Academy of Sciences, Shanghai 201203, China.; 2 Department of Nanomedicine and Theranostics, Institute for Experimental Molecular Imaging, Uniklinik RWTH Aachen and Helmholtz Institute for Biomedical Engineering, Faculty of Medicine, RWTH Aachen University, 52074 Aachen, Germany.; 3 Department of Pharmaceutics, Utrecht Institute for Pharmaceutical Sciences, Utrecht University, 3584 CG Utrecht, The Netherlands.; 4 Department of Targeted Therapeutics, MIRA Institute for Biomedical Technology and Technical Medicine, University of Twente, 7500 AE Enschede, The Netherlands.; 5 University of Chinese Academy of Sciences, Beijing 100049, China.

**Keywords:** Cancer immunotherapy, nanoparticles, immune microenvironment programming, antitumor immune response.

## Abstract

Immunotherapy is rapidly maturing towards extensive clinical use. However, it does not work well in large patient populations because of an immunosuppressed microenvironment and limited reinvigoration of antitumor immunity. The tumor microenvironment is a complex milieu in which the principles of physiology and anatomy are defied and which is considered an immune-privileged site promoting T cell exhaustion. Tremendous research interest exists in developing nanoparticle-based approaches to modulate antitumor immune responses. The increasing use of immunotherapies in the clinic requires robust programming of immune cells to boost antitumor immunity. This review summarizes recent advances in the engineering of nanoparticles for improved anticancer immunotherapy. It discusses emerging nanoparticle-based approaches for the modulation of tumor cells and immune cells, such as dendritic cells, T cells and tumor-associated macrophages, with the intention to overcome challenges currently faced in the clinic. Furthermore, this review describes potentially curative combination therapeutic approaches to provoke effective tumor antigen-specific immune responses. We foresee a future in which improvement in patient's surveillance will become a mainstream practice.

## Introduction

Cancer is being treated by conventional therapies, including surgery, chemotherapy, and local radiotherapy. However, cancer metastasis and relapse lead to therapeutic failure. Cancer immunotherapy has shifted the paradigm by delineating an alternative treatment regimen in order to thwart the development of metastasis. The antitumor immune response can also be augmented by immunotherapies. It can specifically prevent the tumor metastasis as well as relapse by boosting the immune system, modulating the immune response, and inducing immunological memory while minimizing off-target adverse effects 1-8.

Immunogenic cell death (ICD) is associated with a series of well-defined sequential changes, including the constellation of alterations in cell surface of dying cells, such as heat shock proteins and calreticulin (CRT) as well release of soluble mediators, including high-mobility group box 1 protein (HMGB1) and adenosine triphosphate (ATP), which stimulates dendritic cells (DCs) and T cells 9. The receptors on dendritic cells, e.g., toll-like receptor 4 (TLR4), CD91, and P2RX7 can interact with the HMGB1, CRT, and ATP, respectively. The cancer cells translocate CRT to their surface after being exposed to cytotoxic agents. CRT, an “eat-me” signal on tumor surface are recognized by DCs and ultimately trigger ICD. The expression of heat-shock protein 90 (HSP90) on the surface of dying cancer cells can mediate the maturation of DCs 10. The HMGB1 on cancer cells is responsible for processing and presentation of tumor-associated antigens to DCs via TLR4 11, 12. The stressed cancer cells ultimately release the maturation signals (interleukin-1 (IL-1) or interferon (IFN)) leading to the completion of DCs maturation 13. Cancer cells succumbing to ICD are exploited as vaccines to trigger an effective immune response. The antitumor immune response can be initiated by the presentation of tumor-associated antigens (TAA) to DCs. The tumor-associated antigens or neoantigens can be delivered either exogenously as a therapeutic vaccine or ingested *in situ* 14, 15. ICD is obligatorily initiated by various types of stress signals, including chemotherapy and reactive oxygen species (ROS)-induced stress 16, 17. The DCs are then activated by processing and presenting tumor antigens in the context of major histocompatibility complex (MHC). The activated or matured DCs are moved to lymph node in order to elicit T cell-mediated antitumor immune response, especially the generation of CD8+ effector T cells. DCs can also trigger natural killer (NK) cells and antibody production 7. After leaving the lymph node, the effector T cells enter to tumor sanctuary, where immunosuppressive microenvironment may become a challenge 18. The immunosuppressive pathways may impede the effective immune response by depleting T cell lymphocytes 9. On the other hand, ICD is considered one of the major pathways to win the fight against cancer by activating the immune response 9, 13. Therefore, the long-term success of effective treatment lies in immunotherapy.

**Figure 1:**
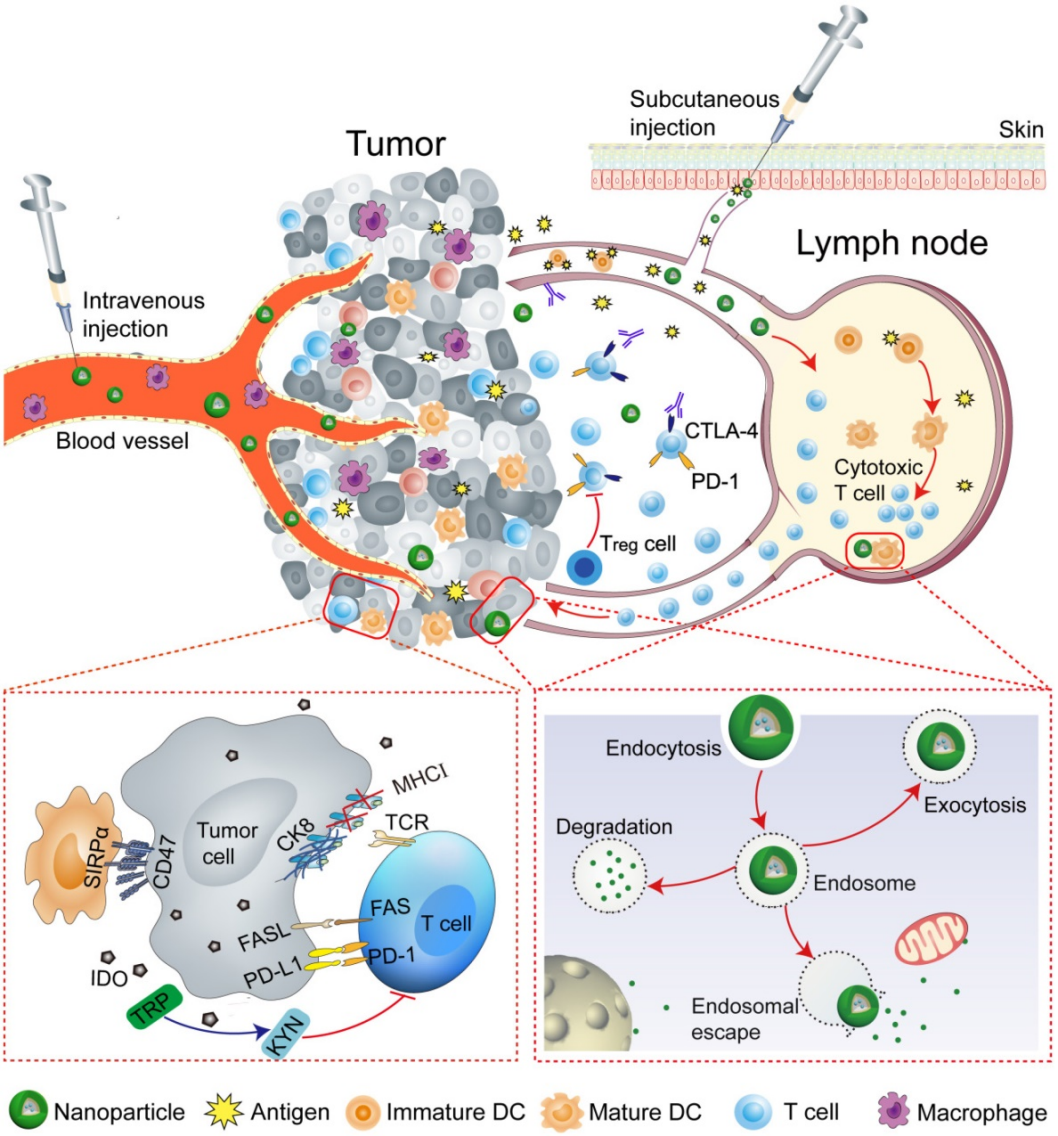
Schematic illustration of nanoparticle-mediated modulation of immune response to improve the effectiveness of cancer immunotherapy. Engineered nanoparticles are taken up and escape the endosome to release a variety of cargoes (such as antigens and adjuvants) in tumor immune microenvironment and lymph nodes whereas the negative regulatory pathways are deactivated by immune-checkpoint inhibitors.

Nanoparticles (NPs) can effectively amplify immunomodulatory effects and modulate the immune response by integrating various molecules. NPs are capable of manipulating immune cells by facilitating the targeted delivery, and can be taken up and escape the endosome to release cargo, including drugs, antigens, and adjuvants at intended target sites (tumor and lymph nodes), while escaping the pathophysiological barriers, such as compact extracellular matrix, endonucleases degradation, and renal clearance, etc. 1, 2, 19-28. Therefore, the curative potential of therapeutic agents can be extended by NP-mediated robust administration. This review will provide an overview of programmable nanoparticles targeting tumor and lymph node to improve the effectiveness of cancer immunotherapy. Nanoparticles that impact various aspects of immunotherapy by precisely targeting specific immune cells, especially DCs, cytotoxic T lymphocytes, tumor-associated macrophages (TAMs) will be discussed. The key strategies that NP-based approaches use to reprogram the tumor microenvironment and immune system are summarized in **Table 1**. The overarching goal of this review is to provide insights into the potential of nanoparticles to improve the outcomes of cancer immunotherapy **(Figure 1)**.

## Nanoparticle-based modulation of the tumor microenvironment

The understanding of the mechanisms underlying the tumor cells to evade immunity as well as to deleterious antitumor immune responses is required to defeat cancer. It is imperative to modulate the tumor microenvironment in order to trigger an effective antitumor immune response by designing NP-based platforms, which have great potential to overcome the pathophysiological barriers.

### Tumor biology and microenvironment

The immune system plays critical 'gatekeeper' functions that are capable of mounting adaptive antitumor response by activating immune cells, especially T cells. However, the tumor microenvironment (TME) is considered the immune-privileged site, which promotes T cells exhaustion by inhibiting their activation and functions 29. The tumor cells are vulnerable to be attacked by the immune system when leaving their sanctuary 30. In hostile environments, the tumor cells may adopt a variety of immune-escape or immunosuppressive mechanisms 18, which promote tumor growth and progression. The cancer immuno-editing are categorized into three stages, such as elimination, equilibrium and escape 31, 32. In the elimination stage, the tumor can be attacked by immune cells. The relation between tumor and immune cells are in equilibrium; however, immune cells may facilitate tumor progression in the equilibrium stage. During the process of tumor progression, the tumor cells ultimately evolve mechanisms of immunosuppression or escape, such as loss or down-regulation of MHC expression as well as up-regulation of different pathways, including programmed death ligand-1 (PD-L1), immune-checkpoint molecules (CD47), and NK-cell ligands (FAS, FAS ligand (FASL)), and thus can actively proliferate in escape phase 31. Therefore, the tumor cells, directly and indirectly, promote immunosuppression by increasing the expression of antagonistic factors and recruiting anti-inflammatory immune cells, respectively 33.

The studies bolstered the concept that immune cells may play a dual role either heroes or villains, where instead of rescue, immune cells support tumor metastasis 18, 34, 35. Regulatory T (T_reg_) cells and myeloid-derived suppressor cells (MDSCs) participate in immunosuppression and tumor metastasis. T_reg_ cells can suppress T cells by releasing transforming growth factor-β (TGFβ) and IL-10 while MDSCs promote immunosuppression by secreting immunosuppressive agents, such as reactive oxygen, arginase, and nitric oxide 20, 36, 37. MDSCs and T_reg_ cells are not the only culprits in tumor metastasis, tumor cells can also exploit other immune cells, especially neutrophils, to “hitchhike” in the process of metastasis 38. There are some immunosuppressive molecules in TME, including indoleamine 2,3 dioxygenase (IDO). IDO suppress T cell functions by degrading essential amino acid, e.g., tryptophan. IDO-expressing DCs may stimulate T_reg_ cells and thus promote the immunosuppression 39. Tumor-associated macrophages (TAMs) as key components in cancer microenvironment play a crucial role in tumor-promoting inflammation and progression. TAMs may either have an inhibitory or supportive influence on cancer depending on disease stage and often dictated by hypoxia 40.

The lymphocytic immune cells, including NK cells, are an integral part of the innate immune system that defend against tumor by adapting different mechanism, such as the receptors-ligands interactions 41. NK cells can induce apoptosis in tumor cells by expressing the tumor necrosis factor (TNF)-related apoptosis inducing ligand (TRAIL) 42. However, tumor cells can escape the immune system via down-regulating the expression of related proteins, including MHC I 43 or adapting inhibitory pathways 44. The tumor cells escape from cytotoxic T lymphocytes (CTLs)-mediated response via loss or down-regulation of MHC I expression 18, 43; therefore, tumor cells evade cell death and facilitate tumor metastasis. The apoptotic pathway, e.g., FAS/FASL is mainly involved in immune evasion. The tumor cells escape from apoptotic cell death via immunosuppressive networks 45, including up-regulation of FASL as well as down-regulation of FAS expression on tumor cells. CD47 on tumor surfaces acts as a “don't eat me” signal and the up-regulation of this antiphagocytic signal enable tumor cells to escape from NK and DC cells while avoiding innate and adaptive immune response 46. Moreover, the adaptation to hypoxia promotes tumor progression 47 and prevents the tumor cells to be attacked by immune cells, since immune functions are impaired in hypoxic niches 48, 49. IDO is one of the major immunosuppressive mechanisms and the aberrant expression of IDO leads to tumor progression 50. The strong correlation between IDO signaling and immunosuppression has been observed. It is generally believed that IDO orchestrates immunosuppression through the recruitment of T_reg_ cells, which ultimately activates MDSCs 50, 51. The reversal of T_reg_ and MDSCs infiltration via IDO inhibitors are found to be an effective strategy to relieve immunosuppression 50-52.

The intrinsic and extrinsic negative regulatory pathways, for example, inhibitory receptors (PD-1), immunoregulatory cell types (T_reg_ cells) and cytokines (IL-10) are involved in T cell exhaustion 53. In human and animal models, the key mechanism of T cell exhaustion or dysfunction is the prolonged up-regulation of multiple inhibitory receptors by exhausted CD8 T cells 54, 55. The negative regulatory pathways can be deactivated by immune-checkpoint inhibitors. Therefore, the immune-checkpoint blockade is considered an effective strategy to relieve immunosuppression, and overcome the anergy of effector T cells by releasing the brakes on antitumor pathways 2, 56-59. Cytotoxic T lymphocyte-associated antigen 4 (CTLA-4) and programmed death protein-1 (PD-1)/PD-L1 blockade are frequently applied strategies in the clinic. CTLA-4 regulates the activation of T cells and therefore T cell-mediated pathway can be activated by blocking the interaction between CTLA-4 and its ligands (CD80 and CD86). PD-1/PD-L1 blockade enables T cell-mediated tumor death by blocking either PD-L1 or PD-1 2, 60, 61. Although the expression of PD-ligands (such as PD-L1 and PD-L2) may differ in humans and mice 62-64, the general idea of T cell exhaustion seems to be same and critical for preventing overt immunopathology 64.

### Engineering nanoparticles to modulate the tumor microenvironment

Nanoparticle targeting platforms have found to be ideal carriers to efficiently co-deliver a variety of cargoes, including drugs, therapeutic peptides, and small molecules. The inherent hallmarks of the tumor microenvironment, such as extracellular matrix, weakly acidic pH, hypoxia, redox potential, etc., have been exploited to design highly targeted delivery systems 65-71. The localized delivery of suitable antagonists and inhibitors can create a favorable environment by activating CTLs. Recently, several groups have explored the great potential of engineered NPs for modulating the tumor microenvironment by overcoming the potential barriers.

**Figure 2:**
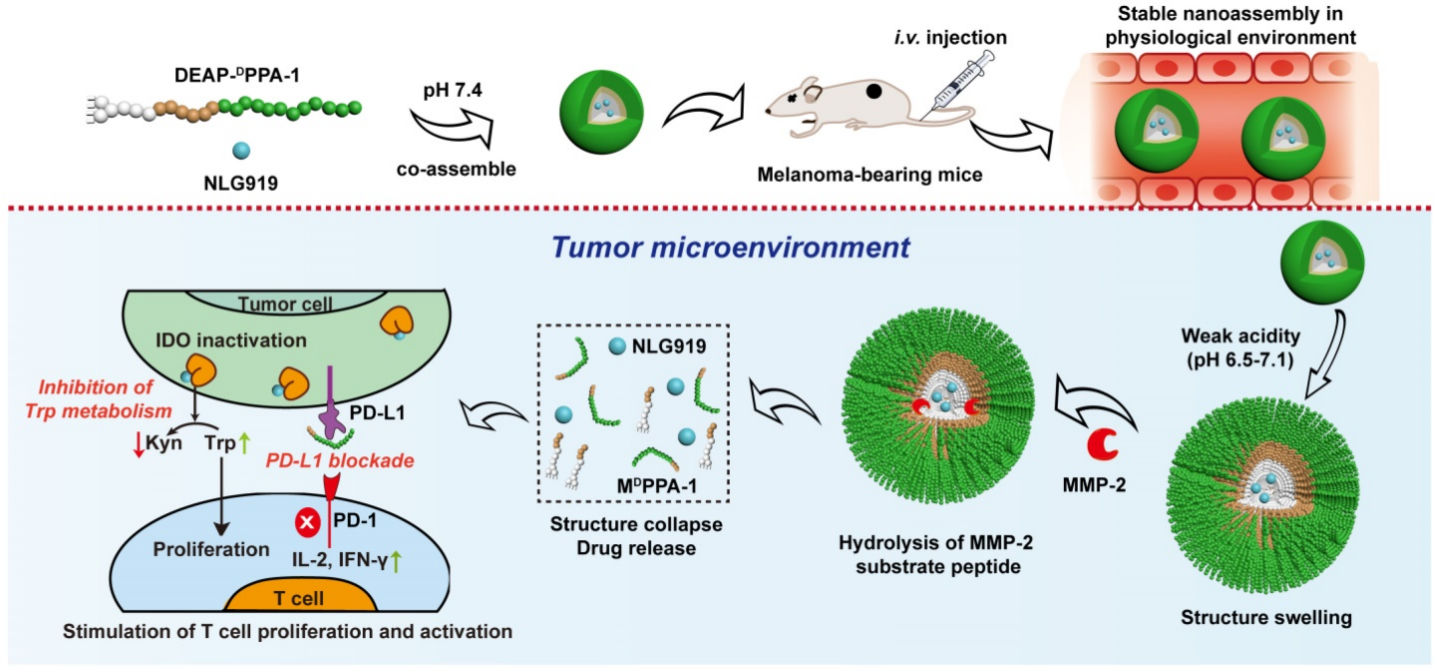
Composition and the proposed antitumor mechanism of the NLG919@DEAP-^D^PPA-1 nanoparticle. DEAP-^D^PPA-1 and NLG919 were co-assembled into a nanoparticle and maintained stable nanostructure at the physiological environment. In acidic tumor conditions, the hydrophobic core of the nanoparticle allowed MMP-2 to hydrolyze its substrate peptide leading to the complete dissociation of the nanostructure. Thereafter, NLG919 and M^D^PPA-1 were released for blocking the immunosuppressive pathways, IDO and PD-L1, respectively. Adapted with permission from 72, copyright 2018 American Chemical Society.

NP-based delivery approaches enabled reversing immune suppression by inhibiting IDO pathway. Guangjun Nie's group designed a peptide-assembled nanostructure (DEAP-^D^PPA-1) containing the hydrophobic and hydrophilic domains, where PLGLAG, a peptide substrate of highly expressed proteinases, e.g., matrix metalloproteinase-2 (MMP-2) and a functional 3-diethylaminopropyl isothiocyanate (DEAP) were linked together to form hydrophobic domain, while hydrophilic domain constituted by a peptide antagonist of PD-L1 (^D^PPA-1). The delivered NLG919, a well-known inhibitor of IDO, co-assembled into micelle-like NPs under physiological conditions. In the acidic tumor niche, the unconsolidated hydrophobic core of NPs allowed MMP-2 to hydrolyze and cleave the peptide substrate, and precisely release modified ^D^PPA-1 and NLG919. The targeted release of modified ^D^PPA-1 and NLG919 ensured the blockade of immunosuppressive pathways, e.g., PD-L1 and IDO, respectively and ultimately rescued CTLs. The level of IL-2 and IFN-γ, as well as the frequency of natural NK cells, were significantly increased after treatment (**Figure 2**) 72, which prolonged survival time by boosting antitumor immune response.

**Figure 3:**
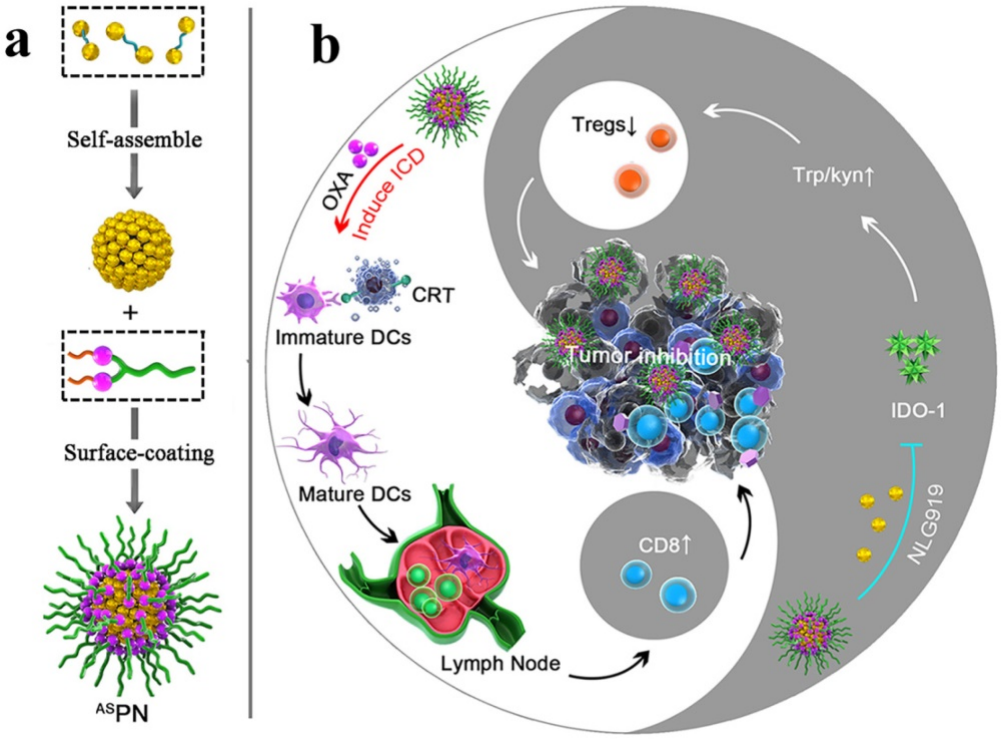
Schematic illustration of the BCPN for improved immunotherapy. (a) Self-assembly procedure of BCPN nanoparticles. (b) Schematic illustration of BCPN capable of modulating the immune response by eliciting antitumor immunity while suppressing regulatory T cells. Adapted with permission from 75, copyright 2018 John Wiley and Sons.

An IDO pathway is considered a key regulator of immunosuppression. Engineered NPs enabled the reversal of immune suppression by blocking IDO pathway. The studies are suggesting a pronounced antitumor effect of the NP-based immunostimulatory formulations 73-75. Feng et al. demonstrated that the tumor microenvironment can be modulated by designing the dual‐activatable (acidic and reducing) binary cooperative prodrug nanoparticle (BCPN), where amphiphilic oxaliplatin (OXA) prodrug and NLG919 were assembled for improved immunotherapy. BCPN ensured improved uptake and penetration due to charge reversal properties of deshielded polyethylene glycol (PEG) in acidic TME, while OXA prodrug and NLG919 were activated in the reducing tumor microenvironment. BCPN can efficiently induce ICD as well as reverse the immunosuppressive pathway owing to the presence of OXA and NLG919, respectively. The obvious increase in tumor cell apoptosis and the decrease in tumor growth were attributed to the activation of DCs, CTLs, and the pro-inflammatory cytokines (IFN‐γ). NLG919 were also found to be effective in inhibiting intratumoral infiltration of T_reg_ cells (**Figure 3**) 75.

Macrophages and DCs are key players of the immune system that are capable of recognizing and presenting antigens to T cells. However, the overexpressing CD47 cancer cells can escape this recognition process by interacting with its corresponding ligand e.g., signal regulatory protein-α (SIRPα) on DCs, macrophages, and neutrophils 46. On the other hand, CD47 blockade drives the phagocytosis of the tumor and T cell-mediated antitumor immune responses 76-78. The NPs can facilitate the reversal of the immunosuppression by modulating tumor microenvironment 79, 80. CaCO_3_ NPs can serve to modulate the acidic tumor microenvironment as well as to precisely release immunomodulatory therapeutics.

**Figure 4:**
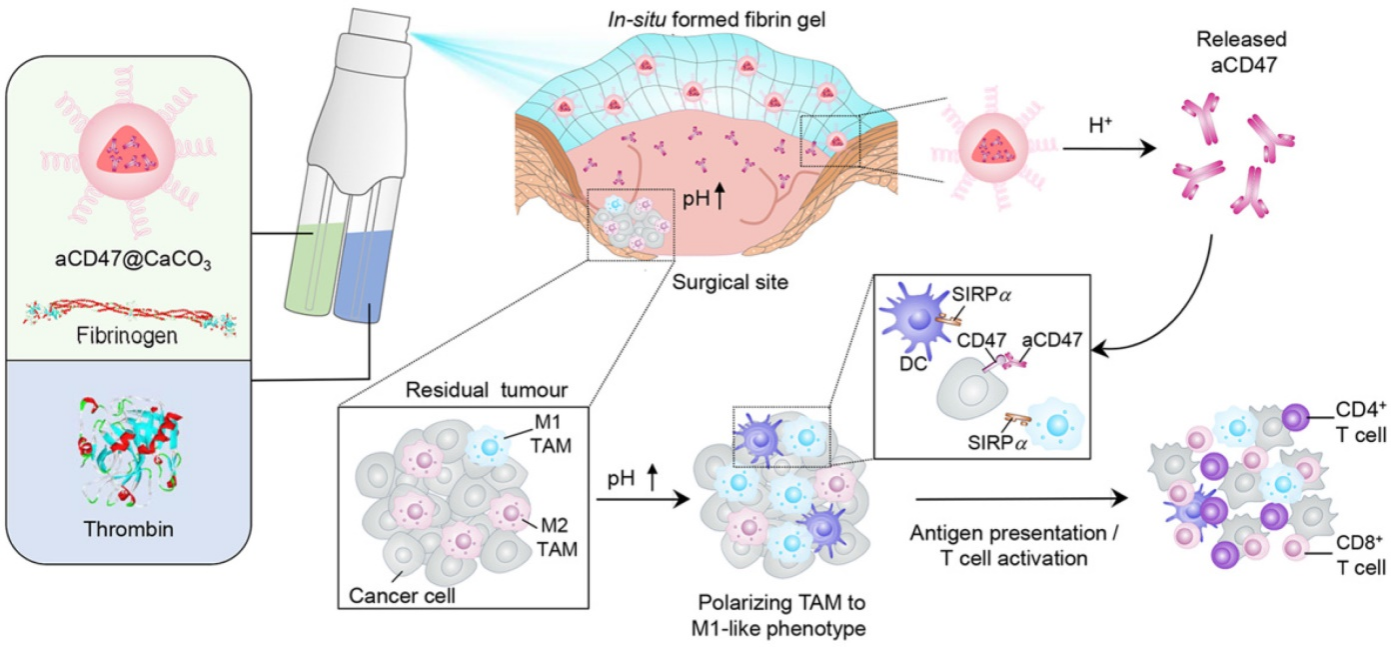
Schematic showing the *in situ* sprayed bioresponsive fibrin gel containing anti-CD47@CaCO3 NPs within the post-surgery tumor bed. Anti-CD47@CaCO3 encapsulated in fibrin scavenged H^+^ in the surgical wound site and precisely released anti-CD47 and thereby promoted the polarization of TAMs (M1-like phenotype) as well as the blockade of the 'don't eat me' signal in cancer cells. Adapted with permission from 80, copyright 2019 Nature Publishing Group.

A recent study reported that the solution of anti-CD47 antibody-conjugated calcium carbonate NPs (anti-CD47@CaCO_3_) can modulate the immune response via blockade of CD47 as well as inducing phagocytosis of cancer cells. CaCO_3_ NPs play an active role in releasing the encapsulated anti-CD47 in the acidic environment by reacting with H^+^ and also act as proton scavenger to increase the pH value. The augmented T cell-mediated immune response and activation of TAMs (M1-type) were observed after treatment. The increased level of IL-12 and decreased level of IL-10 indicated the polarization of macrophages from M2 to M1-like TAMs, which was attributed to NP-based scavenging of H^+^ within the tumor. The immune response was further modulated by overcoming the other potential barriers, such as T_reg_, MDSCs, and hypoxia-inducible factor 1-ɑ (HIF1-ɑ) (**Figure 4**) 80. Engineered NPs can also modulate the immune response by relieving tumor hypoxia 81, 82. The aforementioned studies have provided circumstantial evidence that engineered NPs can promote the recruitment of immune cells in the tumor as well as overcome the anergy of T cells via blockage of immunosuppressive pathways.

## Nanoparticle-based programming of immune cells

The direct tumor targeting yield limited benefits and thus may not attain satisfactory therapeutic index. Therefore, a twist in immunotherapy strategies has been observed in recent years, in which the immune cells are directly targeted instead of targeting tumor cells that are responsible for eliciting an antitumor response. The immune cells are generally considered as “soft target” in comparison to tumor cells, which are secluded behind high interstitial fluid pressure and dense extracellular matrix. Programming of the immune system with engineered NPs has emerged as an effective strategy to improve antitumor immune response. The unique ways in which NPs interact with the immune cells will be reviewed in this section.

### Programming of APCs in lymph nodes

The antigen-presenting cells (APCs) are responsible for initiating T cell-dependent immune responses. Among APCs, DCs are of paramount importance owing to their capability to elicit a T cell-mediated immune response. One of the major challenges in immunotherapy is the lack of activated immune cells in the tumors, especially DCs and T lymphocytes, which are mainly attributed to immunosuppressive pathways. Therefore, the activation of DCs by delivering both adjuvant and antigens in order to induce antitumor immunity, as well as the blockade of immunosuppressive pathways is emerging clinical strategies.

The NPs are being designed to deliver the cargo (such as antigens and adjuvants) through desired intracellular pathways, which allow targeted delivery to the immune cells. NPs have been engineered for lymph node-targeted delivery of various adjuvants, especially pathogen-associated molecular patterns (PAMPs). PAMPs, including cytosine-phosphate-guanosine oligonucleotides (CpG) and R848 can be delivered by NP-based formulations **(Table 1)**. PAMPs are used to trigger immune responses by recognizing pattern recognition receptors (PRR) on the immune cells. Codelivery of antigen and adjuvant by NPs confers enhanced DCs-uptake, antigen cross-presentation, and efficient T cell-mediated response for cancer immunotherapy 84. NPs can target lymph node-residing DCs in a size-dependent manner. Ultra-small NPs (25 nm) can be more efficiently delivered to lymph nodes than large-sized NPs (100 nm) 85. NP-based platforms are believed to be effectively activating immune response while immunosuppressive inhibitor may regulate the therapeutically required dose of the drug among lymphocytes. The delivery of antigen (Ag) and adjuvant to lymph node-resident APCs can be dramatically enhanced by coupling with programmable nanoparticles 86, 87. Therefore, the final key application of NPs is the programming of the immune system in the lymph node.

**Figure 5:**
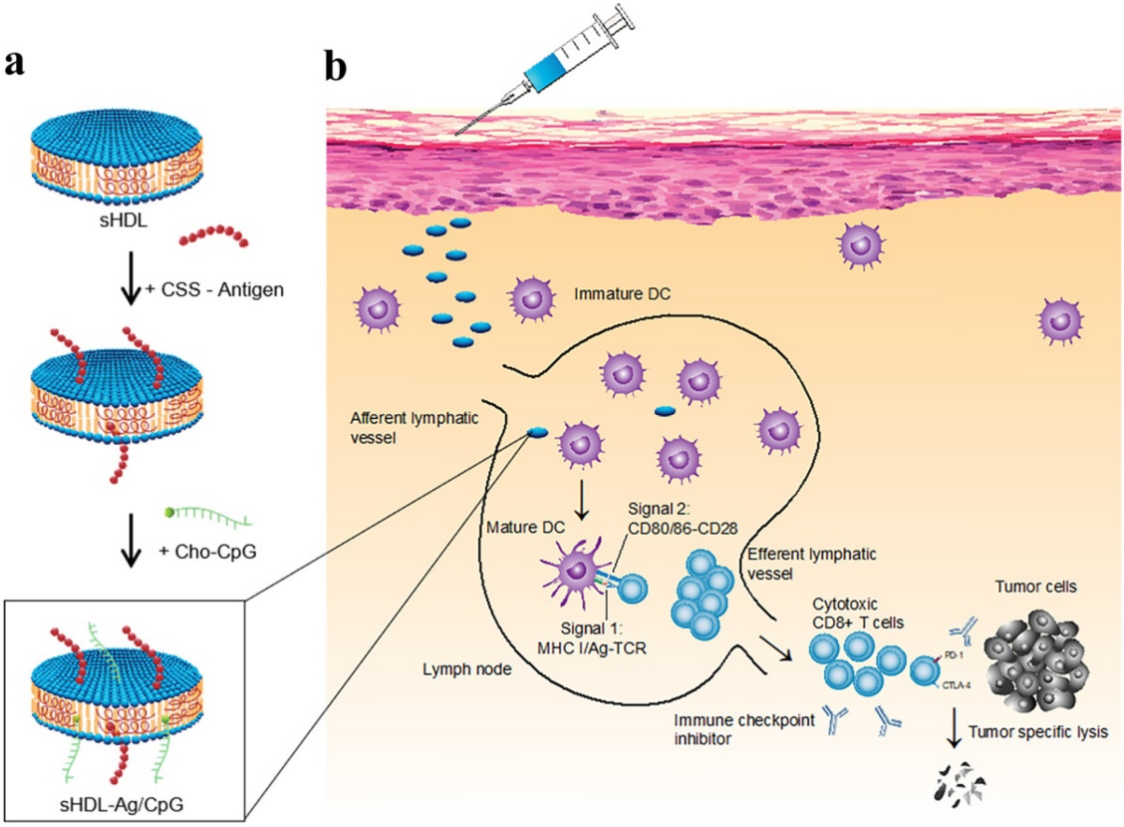
Design of high-density lipoprotein (sHDL) nanodisc platform for personalized cancer vaccines. (a) sHDL nanodiscs, composed of apolipoprotein-1 mimetic peptides (22A) and phospholipids, were engineered to co-deliver antigen (Ag) peptides and adjuvants. The sHDL nanodiscs were mixed with cysteine-modified Ag peptides, including tumor neoantigens that were identified through DNA sequencing of tumor exome and then incubated with a cholesterol-modified immunostimulatory oligonucleotide (Cho-CpG) and subsequent formation of Ag and CpG co-loaded sHDL nanodiscs (sHDL-Ag/CpG). (b) Following administration, sHDL nanodiscs ensured effective co-delivery of Ag and CpG to draining lymph nodes, promoted potent and durable Ag presentation by DCs (Signal 1), and induced DCs maturation (Signal 2), which resulted in stimulation of robust Ag-specific CD8 α+ cytotoxic T-lymphocyte (CTL) responses. Activated CTLs can recognize and kill the targeted tumor cells in peripheral tissues as well as exert potent antitumor responses. Combination immunotherapy with immune checkpoint blockade, i.e., anti-PD-1 and anti-CTLA further improved the efficacy of nanodisc vaccination, leading to the eradication of established tumors. Adapted with permission from 83, copyright 2016 Nature Publishing Group.

A strategy was suggested for augmented CD8α+ cytotoxic T lymphocyte responses in personalized nanomedicine, where Ag/adjuvant co-delivery was markedly improved by conjugating with high-density lipoprotein-mimicking nanodiscs. A nanodisc-based platform was developed by exploiting the intrinsic properties of high-density lipoprotein as a cholesterol nanocarrier and then decorated with tumor neoantigens, i.e., a cysteine-serine serine (CSS) linker coupled Ag peptides. In order to improve cellular uptake and *in vivo* trafficking, a potent TLR9 agonist, i.e., immunostimulatory oligonucleotide (CpG) motif was functionalized with cholesterol (Cho-CpG), and thus designed highly stable, homogeneous, and ultrasmall nanodiscs (sHDL-Ag/CpG). The nanodiscs greatly improved the co-delivery of Ag and adjuvants in lymphoid organs and DCs maturation, which stimulated augmented antitumor T cell-mediated responses to inhibit tumor growth. The cross-priming of T cells could induce multivalent CD4+ T and CD8 α+-mediated T cell immunity. The CTLs frequencies were observed to be 47 and 31-fold higher than soluble vaccines and adjuvant of clinical trials (CpG in Montanide), respectively. However, the antitumor responses could not be sufficient to eliminate the tumor owing to TME induced immunosuppressive PD-L1/PD-1 pathways. Therefore, the immune checkpoint blockade strategy (anti-PD-1 and anti-CTLA) was applied to eradicate the MC-38 and B16F10 tumors **(Figure 5)** 83.

The “albumin hitchhiking” approach, where cargo (antigen or adjuvant) containing amphiphiles-vaccines are linked to albumin-binding tail ensures precise delivery of cargo in the lymph node, T-cell priming, and thereby acquire remarkable antitumor efficacy 88. Based on this concept, it was demonstrated that efficacy of nanovaccines can be remarkably improved by modifying with endogenous carriers, such as albumin that can effectively internalize via endocytosis and thus promote intracellular uptake, Ag processing, and presentation. For co-delivery of peptide Ags and adjuvant into LNs, albumin binding vaccine (AlbiVax) was conjugated with Evan blue (EB) derivatives. The resultant nanocomplexes (albumin/AlbiVax) assembled *in vivo* owing to their binding affinity with endogenous albumin. Albumin/AlbiVax dissociated and liberated AlbiVax in the acidic conditions and thus elicited durable innate and adaptive immunity, and T cell-mediated memory. The therapy efficacy was further improved by combing nanocomplexes with anti-PD-1/Abraxane, which resulted in the eradication of established tumors in multiple tumor models 89.

**Figure 6:**
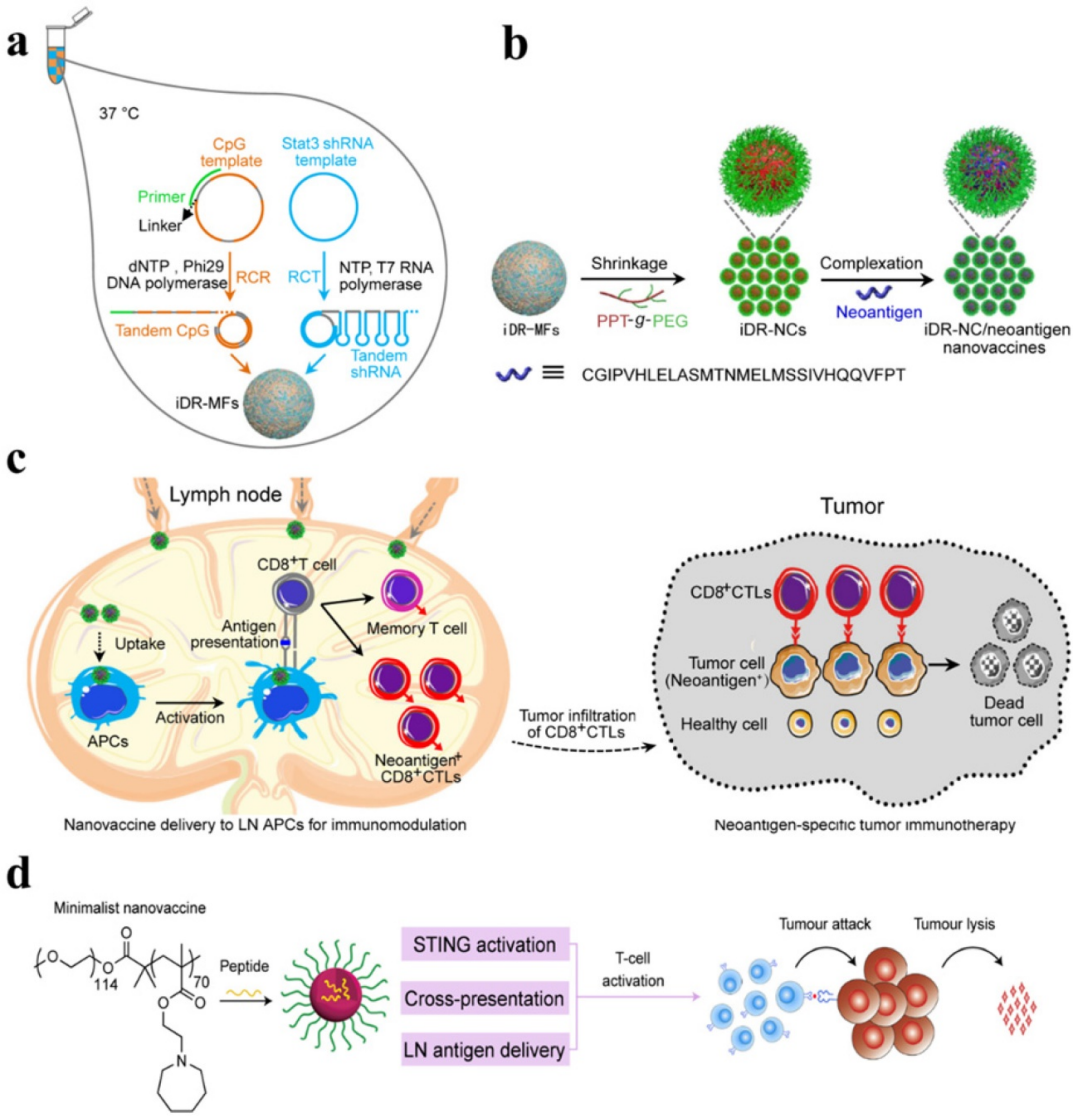
(a) Schematic of iDR-NC/neoantigen nanovaccines for synergistic tumor immunotherapy. The concurrent rolling circle replication (RCR) and rolling circle transcription (RCT) in the same solution producing tandem CpG and STAT3 shRNA, which were self-assembled into intertwining DNA-RNA MFs. (b) The above MFs were shrunk due to the presence of PPT-g-PEG and formed iDR-NCs, which was further loaded with tumor-specific neoantigen via hydrophobic interactions between hydrophobic PPT moieties and peptide antigens. (c) In immunocompetent mice, iDR-NCs/neoantigen complexes were delivered into APCs in draining LNs, which ultimately inhibited tumor growth by eliciting the strong and durable neoantigen-specific T cell responses. Adapted with permission from 90, copyright 2017 Nature Publishing Group (d) Schematic of the minimalist design of the PC7A nanovaccine. PC7A nanovaccine enhanced the antigen delivery, cross-presentation, and activated the STING pathway to robust T cell activation and to boost antitumor immunity for cancer immunotherapy. Adapted with permission from 91, copyright 2017 Nature Publishing Group.

Chen's group and co-workers have designed a self-assembled intertwining DNA-RNA nanocapsules capable of efficiently deliver neoantigens and adjuvants to APCs in lymph nodes and modulating immune response by synergistically targeting signaling pathways (such as TLR9 and signal transducer and activator of transcription3 (STAT3). DNA and RNA therapeutics were integrated into the single nanocomplex to develop biostable nanovaccines. CpG, STAT3-silencing short hairpin RNA (shRNA), and tumor neoantigens were incorporated in the same reaction system and self-assembled via rolling circle replication and transcription to form microflowers (MFs), termed as iDR-NC/neoantigen complexes (iDR-NC-MFs). The iDR-NC-MFs having shrinking capability due to the presence of the biocompatible PEG-grafted polypeptide (PPT-g-PEG) and therefore shrunk into iDR-NCs in acidic endolysosomes, and exhibited the enhanced proton sponge effect to facilitate cytosolic delivery of the cargo at the intended target. iDR-NCs also acquired tumor-specific neoantigen loading capabilities. The targeted delivery of iDR-NCs/neoantigen elicited APCs for sustained antigen presentation, about 8-fold more frequent neoantigen-specific CD8+ T cells than CpG and ultimately eliminated colorectal tumors by acquiring strong and durable antitumor immunity **(Figure 6 a-c)** 90.

The stimulation of potent tumor-specific T cells is considered as one of the major challenges in cancer immunotherapy. Therefore, the researchers explored the different synthetic compositions in an attempt to achieve a robust T-cell response. It is proposed that immunotherapeutic armamentarium can be expanded by designing stimulator of interferon genes (STING) pathway-activating NPs to improve the clinical performance of cancer immunotherapy 91, 92. To activate a STING pathway, a simple mixture of an antigen and synthetic polymeric NPs (PC7A NP) is introduced, which can generate a potent cytotoxic T-cell response while minimizing systemic cytokine expression. PC7A NP delivered tumor antigens to APCs in lymph nodes and activated DCs maturation. Type I interferon pathways can be activated by TLR and STING. However, PC7A NPs are observed to be responsible for the activation of type I interferon-stimulated genes via STING. As a result, the increased expression of CD86, the augmented antigen-specific CTL, Th1, and Th2 responses as well as antitumor immunity was developed. The T cell activation may be affected by the expression of IDO-1. Therefore, PC7A nanovaccines with checkpoint inhibition (anti-PD-1) are reported to be a general strategy to overcome potential barriers. As a result, the survival rate is reported to be increased up to 100% in a TC-1 tumor model **(Figure 6d)** 91. Fan et al. proposed a strategy to exploit the immunogenically dying tumor cells for cancer vaccination, where dying tumor cells may act as a source of antigens and danger signals. The binding affinity between the free sulfhydryl on immunogenically dying tumor and maleimide-displaying CpG-NPs were utilized to deliver the adjuvants, which resulted in DCs maturation, antigen-specific T cells and antitumor responses, and ultimate eradication of established CT26 tumor in ∼78% of mice when combined with anti-PD1 therapy 93. DCs have been precisely targeted in lymph nodes by using lipid carrier without further functionalization. The systemically administered RNA-lipoplexes (RNA-LPX) vaccines successfully delivered the encoded antigens in lymph nodes, bone marrow, and spleen and elicited a potent type-I-IFN-mediated immunostimulatory response 94.

Mesoporous silica rods (MSRs)-based formulations have been demonstrated for the recruitment of DCs in lymph nodes and augmented systemic helper T cells response in order to modulate immune function and elicit adaptive immune responses 95, 96. A study reported a simple and powerful multi-antigen approach to improve the therapeutic potential of a cancer vaccine, where a polyethyleneimine (PEI) was simply adsorbed in mesoporous silica microrod (MSR) without any peptide modification, and thus the properties of PEI were exploited to deliver an antigen in MSR vaccine. This platform exhibited improved performance in multiple tumor models, where E7 peptide containing MSR-PEI vaccine could eradicate established TC-1 tumors in 80% of mice while inducing immunological memory. MSR-PEI vaccine could also be combined with anti-CTLA4 therapy for the effective eradication of established lung metastases 96. Moreover, iron oxide-zinc oxide core-shell NPs have been demonstrated as an effective carrier for delivering carcinoembryonic antigen into DCs and provoking antigen-specific T cell responses 97. Gold nanoparticles have also been reported for the delivery of TLR7 immunostimulant drug (R848) to tumor-draining lymph nodes 98. Ovalbumin (OVA)-coated pegylated poly(lactic-co-glycolic acid) (PLGA) NPs with TLR3 and 7 ligands, PLGA-(Ag/TLR3+ 7L) NP 99, encapsulin (Encap) incorporating genetically engineered OT-1 peptides of OVA, (OT-1-Encap) 100, and OVA-conjugated polymeric micelles 101 have been explored in an attempt to get improved immunotherapeutic performance. Several strategies have been perused to targeting the lymph node. It seems logical, therefore, that the capability of NPs is of particular importance to transport the cargo, and thus promote the modulation of the immune response.

### Programming of T cells

The effective stimulation of T cells to defeat tumor is the ultimate goal of cancer immunotherapy 102. The adoptive cell transfer therapy, including chimeric antigen receptor therapy (CAR T) can be a promising method in this context 103, 104; however, the limited response rate, laborious manufacturing processes, and lack of efficient procedures are some of the main limitations in this method. NP-based programming may overcome the critical limitations by simplifying the expansion process 1, 105.

**Figure 7:**
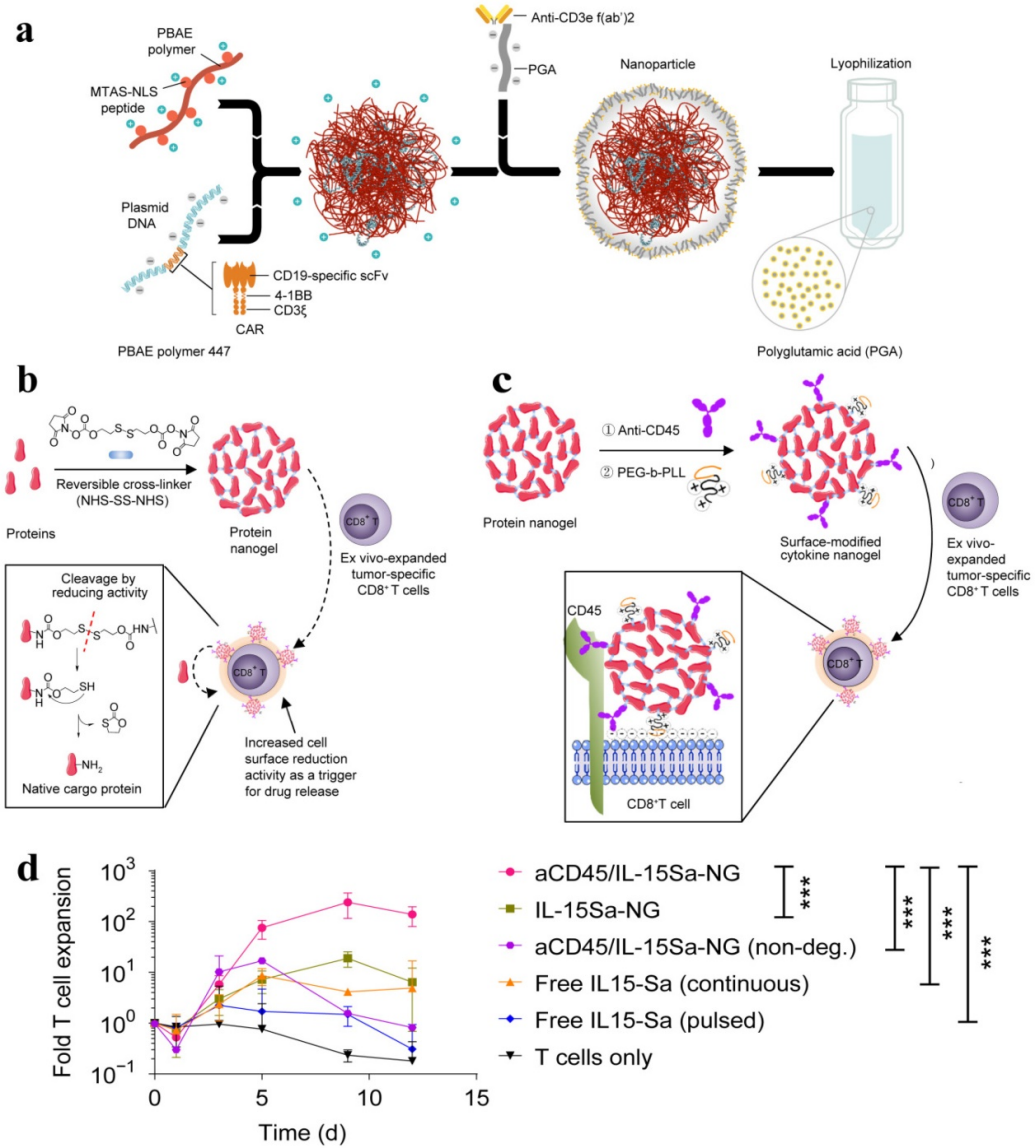
(a) Schematic of the T cell-targeted DNA nanocarrier. The fabrication of NPs is demonstrated. The two plasmids encoding for 194-1BBz CAR and the iPB7 transposase were encapsulated into the nanoparticles. Adapted with permission from 105, copyright 2017 Nature Publishing Group. (b) Schematic of the synthesis of protein NG and protein release in response to reducing conditions of local microenvironment. (c) Scheme for surface modification of cytokine-NGs that ensured effective and stable anchoring on T cell surfaces. (d) Fold expansion of naive CD8+ T cells that were stimulated with anti-CD3/CD28 beads in the presence of surface-bound aCD45/IL-15Sa-NGs, IL-15Sa-NGs or nondegradable NGs (aCD45/IL-15SaNGs(non-deg.)) or that were incubated with free IL-15Sa. Adapted with permission from 106, copyright 2018 Nature Publishing Group.

The lack of cytotoxic T lymphocytes as well as regulation of the primed T cells is one of the main challenges of immunotherapy. NP-based DNA carriers can precisely program tumor-recognizing capabilities into circulating T cells and thus capable of inducing tumor regression. A simplified, cost-effective, and practical programming approach was introduced to generate “on demand” antitumor immunity, where polymeric nanocarriers functionalized with lymphocyte-targeting ligands precisely delivered leukaemia-specific CAR genes into T cells 105. To achieve targeted DNA delivery into host T cells, the surfaces of NPs (biodegradable poly(β-amino ester)) were conjugated with T cell-targeting fragments, i,e., anti-CD3e f(ab′)2, which ensured receptor-mediated endocytosis. NPs were functionalized with nuclear localization signals (NLS) and microtubule-associated sequences (MTAS), which enabled the import of DNA into T cell nucleus by employing the microtubule transport machinery. The anticancer programming capabilities were induced by decorating the targeted nanoparticles with a plasmid DNA encoding fusion receptor, such as leukaemia-specific 194-1BBz CAR having single-chain antibody (scFv), 4-1BB, and CD3ζ domains, which are most frequently used to target CD19 in clinics **(Figure 7a)**. CD19-specific CARs genes containing NPs were incubated with T cells *in vitro* whereas intravenously delivered to re-program T-cell *in vivo*. CAR did not fully express and induces such programming in other cells and therefore leukemia regression was effectively achieved *in vivo* (comparable to conventional adoptive transfer CAR T). Reprogrammed T cells acted as a “living drug' to eradicate the tumor, and induced immunological memory since the expressions of these receptors were maintained even for weeks.

It was demonstrated that the performance of T cell therapy can be reprogrammed by T cell receptor (TCR)-activatable NP drug delivery system. T cells are capable of modulating cell surface redox state when activated due to the transformed expression of their reducing enzymes. This discovery was exploited to achieve antigen-triggered release of adjuvant protein by designing reduction-responsive NPs. The activated protein nanogels (NGs) in T cell surface reduction potential were employed to 'backpack' supporting cytokines on T cells. The supporting cytokines, such as human interleukin 15 super-agonist (IL-15Sa) was selected as a drug cargo in this context. The disulphide cross-linker was cleaved by reducing conditions of T cell surface and released the cargo, i.e., intact cytokine in antigen-bearing microenvironments. The reducing conditions of TME, including glutathione, can also promote the release of IL-15Sa from NGs. However, T cell surface redox-mediated cytokine release was observed. A small quantity of anti-CD45 and poly(ethylene glycol)-b-poly(L-lysine) (PEG-PLL) was incorporated into the NGs, where anti-CD45 kept the NG backpack intact on the surface by preventing internalization and PEG-PLL maximized the NG loading efficiency by providing a uniform positive charge **(Figure 7b-c)**. The redox-responsive anti-CD45/IL-15Sa-NGs stimulated augmented T cell expansion *in vitro* **(Figure 7d).** The anti-CD45/IL-15Sa backpacked T cells were intravenously injected in mice. About 8-fold higher cytokine dose and 16-fold specifically expanded T cells was achieved by programming the location and timing of T cell activation, which ultimately resulted in improved tumor inhibition 106.

The conjugated supporting drugs, including antibodies and interleukins, can secure T cells from immunosuppression 107. To specifically target the CD8+ T cells in lymphoid tissues, NPs are functionalized with antibody (anti-CD8a F(ab')2 fragments). The main mediator of immunosuppression, e.g., TGFβ can be targeted by TGFβR1 inhibitor (SD-208) in order to rescue the suppressive immune cells, to activate the CD8+ T cells, and to restore the activity of effector T cells. NP-based targeted delivery of a TLR7/8 agonist (R848) and immunosuppressive inhibitor (SD-208) can greatly improve therapeutic index, where R848 can activate T cells while SD-208 ensure an appropriate dose of the drug among lymphocytes. The targeted delivery of SD-208 or R848 by the PD-1-targeting NPs therefore reported to inhibit tumor growth whereas free anti-PD-1, SD-208 or R848 could not inhibit tumor growth 108. Reprogrammed T cells have also been demonstrated as ideal carriers to improve cellular uptake by overcoming the drug delivery barriers. The homing receptors expressing T cells were exploited as nanoparticle carriers. The potent topoisomerase I poison SN-38 containing lipid nanocapsules (NCs) were covalently loaded to the cell membrane of primary T cells without affecting the expression of surface receptors. SN-38-releasing NCs systemically delivered 90-fold greater drug into lymphoid organs than free drug in order to specifically target lymphoma cells 109. Thus, the delivery efficiency of SN-38 can be greatly improved by T cell-mediated delivery systems, which result in more effective tumor inhibition and enhanced survival than the free drug. Taken together, these studies exemplify the success of NP-based programming of T cells to remarkably modulate the T cells response. However, special attention should be paid to achieve a safe design in order to avoid deleterious outcomes since the aberrant T cell functions may lead to autoimmune diseases 110.

### Reprogramming tumor-associated macrophages

Macrophages are one of the major components of the leukocyte infiltrate that is engaged with cancer in a dual yin-yang relationship by exhibiting M2-M1 phenotypes. M1-like phenotypes are correlated with antitumor responses and mainly driven by bacterial products and IFN-γ whereas M2-like phenotypes driven by IL-4 or IL-13 are responsible for tumor progression and adaptive immune suppression 111, 112. IFN-γ is mainly responsible for the polarization of macrophages by stimulating the transcriptional network, epigenetic, and metabolic pathways. IFN-γ-polarized macrophages are hypersensitive to various pro-inflammatory stimuli, including TNF, type I interferons, lipopolysaccharide, and ligands of TLRs. TLRs-mediated polarization of macrophages results in the activation of nuclear factor-κB (NF-κB) target genes and inflammatory cytokines. Moreover, IFN-γ can induce resistance to anti-inflammatory stimuli, including IL-10, IL-4, and IL-13 and thus promote the polarization 113.

**Figure 8:**
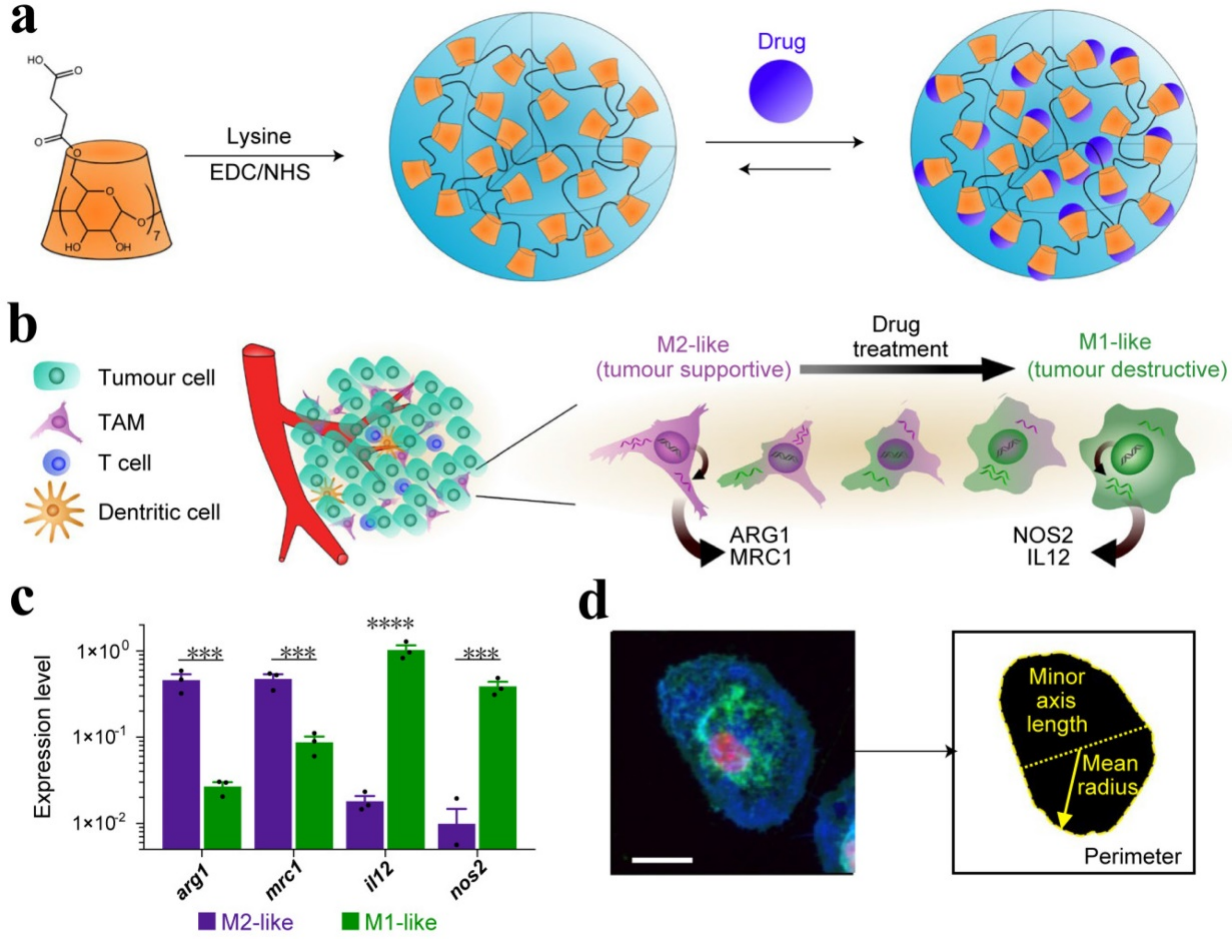
(a) Schematic of CDNP preparation by lysine crosslinking of succinyl- β-cyclodextrin (orange) and subsequent drug loading by guest-host complexation of R848 (blue). (b) Schematic overview of the tumor microenvironment, where TAMs were mainly canonically M2-like; however, their behavior was pharmacologically influenced. (c) Gene expression of M2-like (IL-4 treated) and M1-like (LPS/INF-γ treated) murine macrophages. (d) Raw images were processed by automated segmentation for the measurement of prominent features useful in the identification of M1-like polarization as indicated in yellow, where the mean radius (solid line), minor axis length (dotted line) and perimeter (dashed line). Cells were stained for nuclei (DAPI, red), actin (phalloidin, green), and cell membrane (WGA, blue). Scale bar, 25 μm. Adapted with permission from 114, copyright 2018 Nature Publishing Group.

TME comprised of a variety of cells, including TAMs, which play a critical role in the immune system. A continuum of macrophage phenotypes, such as M1 and M2 exists. The recent studies suggest that macrophages contribute to the immunosuppression in the TME while the reprogramming of TAMs from M2 to an antitumor M1 mode and inhibition of macrophage recruitment can provoke macrophage-mediated extracellular cancer death. Therefore, the polarization of TAMs or re-programming is considered a clinically approved therapeutic strategy 40, 111. The increased expression of metabolic checkpoint enzyme arginase-1 (ARG1) and mannose receptor-1 (MRC1) are triggered by IL-4 treatment in M2-like signature (tumor supportive) while the higher expression of IL-12 and nitric oxide synthase (NOS2) is associated with M1-like (anti-tumor) signature, which may be induced by IFN-γ secretion. Cell morphology is considered as a tractable biomarker of cell function. The analysis of macrophage state, therefore, provides a simplified model to assess the M1 or M2 signature. M1-like state exhibit flattened and round morphology in contrast to M2-like cells that display elongated projections. In order to reprogram the TAMs, Weissleder and colleagues introduced a targeting approach utilizing small molecules. Dextran and cyclodextrin (CD)-based NPs are preferentially distributed to TAMs owing to their intrinsic macrophage avidity. The native macrophage avidity was therefore exploited. An agonist of the TLR7 and TLR8 (R848) were demonstrated as a potent driver of the M1 phenotype in this context. The R848-loaded β-cyclodextrin NPs (CDNPs) was designed by leveraging the interaction between CD and R848, where the amide bond formed between succinyl- β-cyclodextrin and l-lysine. TAM-targeted CDNPs efficiently delivered R848 to TAMs in the TME and induced adaptive immune responses, including changes in myeloid phenotype and the ultimate increase in IL-12 level. The analysis reflected the obvious changes in cell morphology and significant enrichment in M1-like cells as compared to M2 **(Figure 8)**. CDNP-R848 re-educated the TAM phenotype and thus ensured the reduced tumor and improved survival rate in CDNP-R848 treated mice after a single dosage. The synergistic treatment with anti-PD-1 further improved the therapeutic potential, which resulted in complete tumor regression in 2/7 mice while inducing antitumor memory 114.

Macrophage reprogramming has also been demonstrated by Saeid Zanganeh using iron oxide NPs (ferumoxytol) and suggested that ferumoxytol could be applied 'off label' to suppress metastasis as well as to improve the performance of macrophage-modulating cancer immunotherapy approaches 115. In the tumor microenvironment, TAMs can easily engulf the administered NPs and this fact was exploited to deliver ferumoxytol NPs. After being internalized, ferumoxytol induced pro-inflammatory immune response and polarization of M1 macrophages. Pro-inflammatory M1 macrophages can release hydrogen peroxide and therefore react with iron via the Fenton reaction and ultimately generate hydroxyl radicals (OH^•^), which can induce apoptosis in the cancer cell. Therefore, the increase in caspase-3 expression and pro-inflammatory Th1-type responses was observed. Compared to control groups, about 11 and 16-fold higher level of hydrogen peroxide and hydroxyl radicals was observed in macrophages plus ferumoxytol-treated cells, respectively. Moreover, the increased expression of M1-related markers, i.e., TNF-α and CD86 and the decreased level of M2-related markers, such as IL-10 and CD206 after exposure to ferumoxytol, suggested induction of M1 macrophage polarization. This study demonstrated that tissue-resident macrophages could lose their M2 polarization while infiltrating macrophages was shifted towards M1 polarization. Ferumoxytol is therefore effective in inhibiting pulmonary metastasis and established liver tumors in a TAM-dependent manner. The NP-based gene-editing platform can also be designed that are able to reprogram immune responses. NLRP3 inflammasome, one of the endogenous damage-associated molecular patterns for the treatment of multiple inflammatory diseases can be targeted by using NPs. Cationic lipid-assisted nanoparticle (CLAN), a type of PEG-b-PLGA-based NPs has been demonstrated as a carrier for delivering Cas9 mRNA that guides RNA into macrophages and ultimately disrupts NLRP3. CLAN is capable of selectively targeting and reprogramming the macrophages since NLRP3 specifically function in macrophages 116. Thereby, this strategy avoids systemic side effects and may be safely implemented for the treatment of inflammatory diseases, including cancer.

Angiogenic factors, including vascular endothelial growth factor (VEGF), are highly expressed in TAMs and responsible for tumor progression and metastasis. A strategy presented by Conde et al. to reprogram TAMs where targeting immunotherapy was combined with silencing in the tumor microenvironment. Gold NPs having a peptide sequence (M2pep) selectively targeted TAMs and delivered a small interfering RNA (siRNA) to silence the expression of VEGF mRNA in the inflammatory M2 macrophages, which could transform immune response from an immunosuppressive to an immunostimulatory as well as tumor inhibition in lungs 117. Huang et al have designed a trastuzumab-modified, mannosylated liposomal system (tLGV) for targeting the overexpressed receptors in TAM2 and reprogrammed the M2 polarization to M1 by downregulating T790M mutation in epidermal growth factor receptor (EGFRT790M) 118. This section has highlighted the potential of NP-based formulation to directly target the immune cell, such as TAM in the tumor microenvironment, which can effectively boost local and systemic antitumor immunity.

## Combination therapies

The cancer relapse even after combination conventional therapies, such as surgery, radiotherapy, and chemotherapy leads to therapeutic failure. It is imperative to introduce alternative strategies to defeat cancer. The focus of alternative strategies should be on the stimulation of immune response, which will induce immunological memory to overcome the cancer relapse 13. Damage-associated molecular patterns (DAMPs) are surface-exposed, released or secreted by dying/stressed cells. DAMPs, such as surface-exposed CRT, passively released HMGB1, secreted ATP, and heat-shock proteins that can act as either danger signals or adjuvant for the immune system to induce ICD in cancer cells 9, 16, 119, 120. As cancer cells die, they release DAMPs that can act like a signal or combinatorial code to unlock various immune responses 121. ICD inducers, including chemotherapeutic agents (anthracyclines and oxaliplatin) and photodynamic therapy (PDT), can trigger immunogenic apoptosis in cancer cells, where dendritic cells engulf their corpses and present tumor-specific antigen to T cells to provoke antitumor immune response 17.

ICD can be induced by the generation of ROS and endoplasmic reticulum (ER) stress by instigating the danger signaling pathways. The stress-specific mechanisms are involved in releasing key DAMPs, such as CRT and ATP 16. Therefore, PDT can induce ICD in cancer cells by generating ROS-based ER stress 122. Anthracyclin-treated cancer cells can also induce ICD and elicit the antitumor immune response without any adjuvant. The cancer cells that are undergoing ICD can trigger the translocation of CRT to their cell surface, which ultimately leads to tumor antigen-specific cytotoxic T lymphocyte-mediated immune responses 123, 124. Although the PDT and chemotherapy can initiate an antitumor immune response, the induced immune response is severely impaired by T cell exhaustion through the up-regulation of PD-1/ PD-L1. T cell exhaustion can be reversed by blocking immune checkpoint pathways (such as PD-1) 125, 126. Immunotherapy with immune checkpoint blockade can be used to expand the frequency and functionality of tumor-specific T cells to potentiate antitumor efficacy 127, 128.

NP-based strategies can be applied to exploit the ICD-inducing properties of conventional therapies for improving the therapeutic potential of cancer immunotherapy. NP-based combination therapies, including photothermal therapy, photodynamic therapy, radiation therapy, and chemotherapy with immunotherapy improve the therapeutic performance by enabling the simultaneous delivery of various therapeutic molecules 20, 129-134.

**Figure 9:**
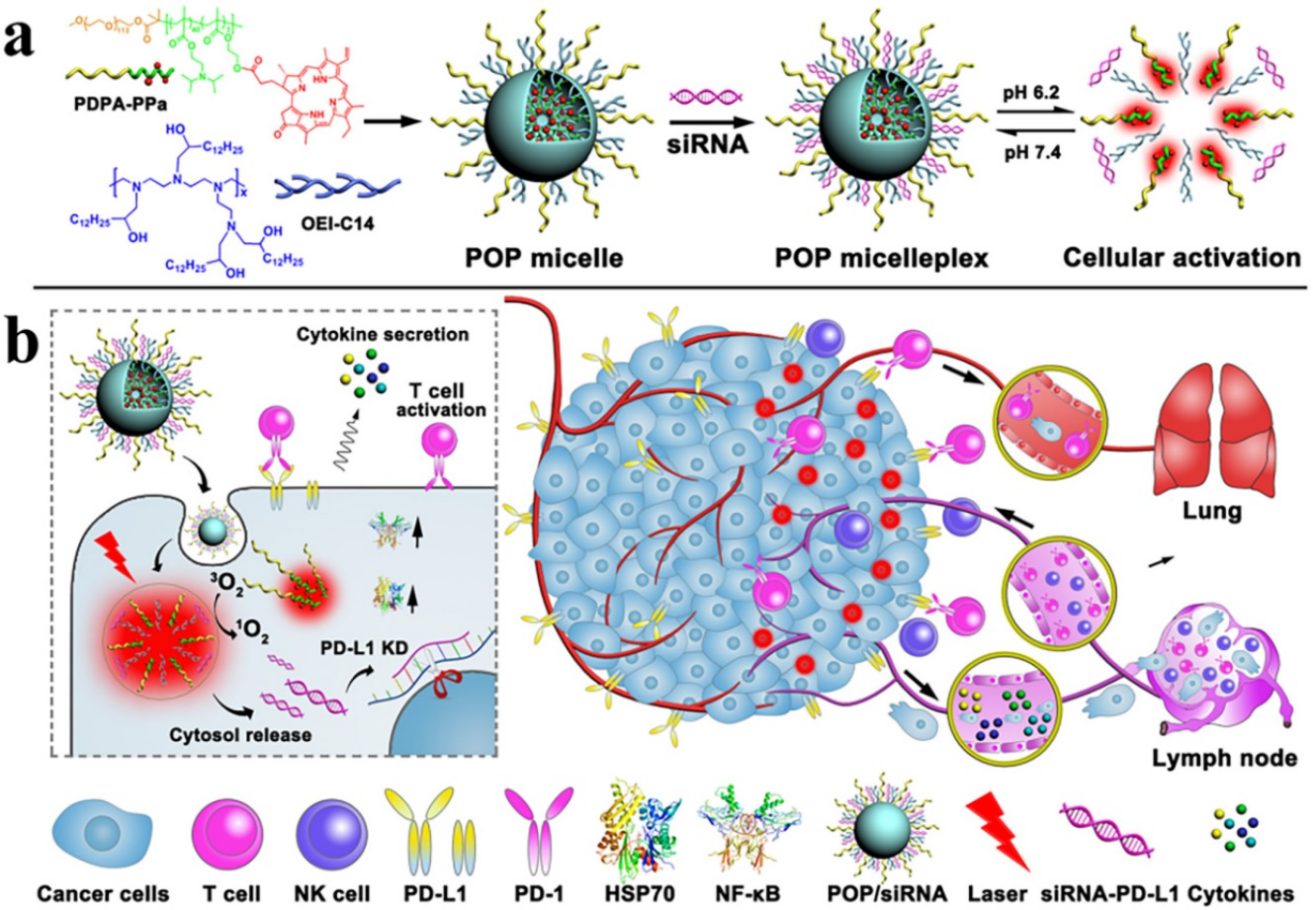
Schematic Illustration of the acid-activatable micelleplexes for PD-L1 blockade-enhanced photodynamic cancer immunotherapy (a) Chemical structure of the acid-activatable POP micelleplexes that were co-loaded with PPa and siRNA; the micelleplexes dissociated at an acidic microenvironment that were attributed to the protonation of the of PDPA. (b) Schematic of POP-PD-L1 micelleplex mediated combined cancer immunotherapy. Upon PDT, the POP-PD-L1 micelleplexes generated ROS, which ultimately induced an adaptive immune response by provoking HSP70 and NF-κB pathways, triggered the secretion of the pro-inflammatory cytokine, and recruited tumor-infiltrating T cells. The antitumor immune response was further improved by RNAi of PD-L1. Adapted with permission from 135, 2016 American Chemical Society.

Wang et al. designed a versatile micelleplex, which could enhance the therapeutic potential by combing multiple functionalities, such as ultra- pH-sensitive diblock (PDPA), pheophorbide A photosensitizer (PPa), and siRNA. The conjugated amphiphilic polycation, e.g., 1,2-epoxytetradecane alkylated oligoethylenimine (OEI-C14) having an intrinsic binding affinity with siRNA and thereby ensured the endosomal escape of the siRNA by promoting proton sponge. PDPA-OEI-C14-PPa (POP) micelleplex was precisely activated in acidic pH (6.2) while keeping intact in extracellular microenvironment. The siRNA-PD-L1-conjugated micelleplexes (POP-PD-L1) effectively induced PD-L1 blockade and thus rescued from immunosuppression by silencing PD-L1 expression on the tumor cells. The antitumor immune response was amplified by photodynamic therapy (PDT), where it effectively eradicated the tumor and distant metastasis in B16-F10 melanoma model by promoting cytokine secretion (TNF-α and IFN-γ) and the frequency of the tumor infiltrating lymphocytes (CD8+ and CD4+). The more apoptotic cancer death was observed after combination therapy than individual modalities and thus ultimately induced immunological memory owing to the presence of activated immune cells (**Figure 9**) 135.

**Figure 10:**
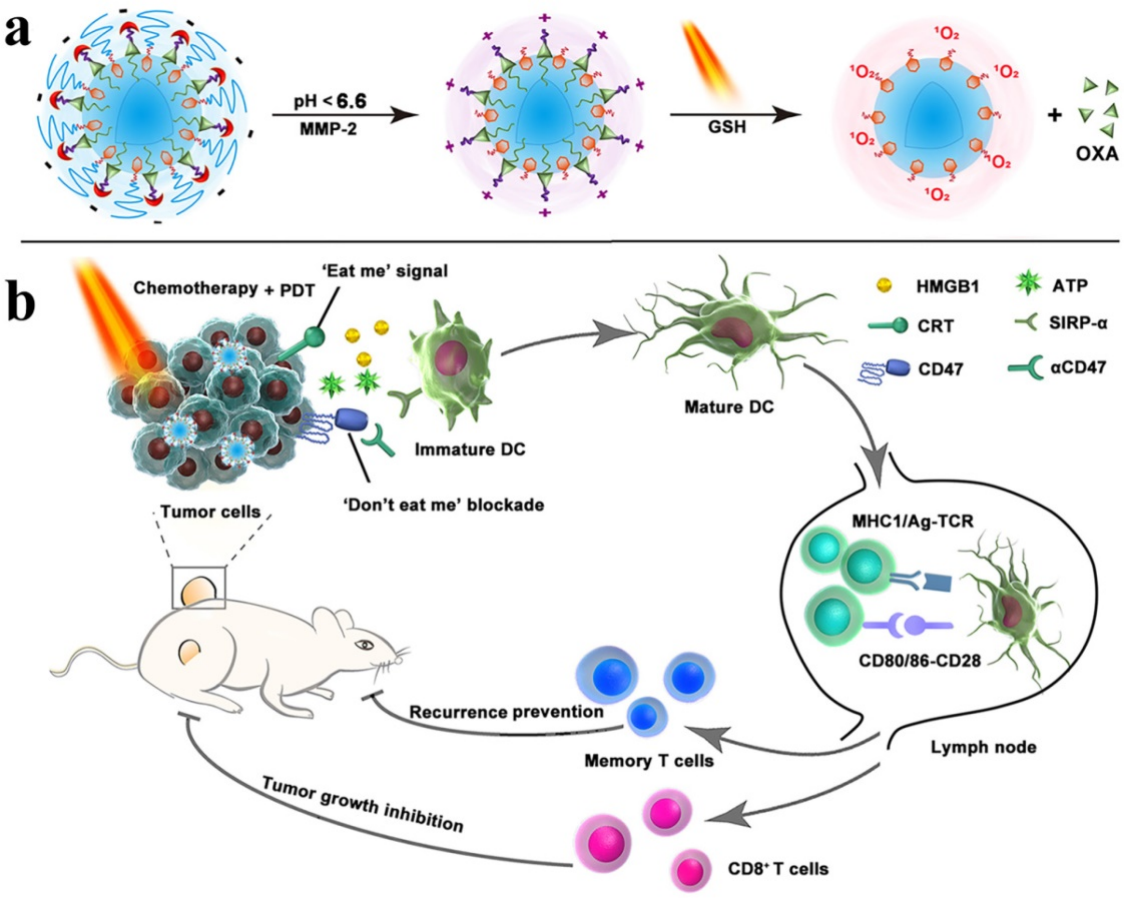
Schematic illustration of the prodrug vesicles for improved cancer immunotherapy by combing ICD induction and CD47 blockade. (a) Schematic design of the dual-responsive, e.g., acidity and MMP-2 prodrug vesicles. (b) Simplified mechanism of NP-mediated chemo-immunotherapy and CD47 blockade, which inhibited tumor growth, distant metastasis, and relapse. Adapted with permission from 140, copyright 2019 John Wiley and Sons.

The doxorubicin-loaded lipoprotein-mimicking nanodiscs (sHDL-DOX) can exert antitumor efficacy by triggering ICD of cancer cells. Combination chemoimmunotherapy can sensitize tumor cells to immune checkpoint blockade and potentiate antitumor T cell-mediated responses. Elimination of established tumors (MC38 and CT26 colon carcinoma) in 80-88% of animals was achieved by delivering chemotherapeutic agents via nanodiscs. Survivors were protected against tumor relapse due to the induced antitumor memory 136. ICD-inducing chemotherapeutic agent, including doxorubicin, oxaliplatin, and cisplatin were delivered by NP-based formulations 137-139, which resulted in synergistic immunotherapy responses when combined with IDO inhibitor (indoximod) or immune checkpoint blockade 137, 138. Various therapies, including chemo-PDT therapy, are not effective against metastasis; however, the combination of chemo-PDT therapy with checkpoint-blockade therapy can not only inhibit tumor growth but also exhibit promising results against tumor metastasis due to the reversal of T cell exhaustion. The chlorine e6 and doxorubicin-loaded hollow manganese dioxide nano platform (H-MnO_2_-PEG/C&D) can relieve tumor suppression whereas checkpoint-blockade (PD-L1 blockade) promotes the higher TNF-α secretion and augmented CD4+ and CD8+ T cells-mediated immune response than chemo-PDT therapy 81. For improving the performance of immunotherapy, Zhou et al. designed a nanoplatform by integrating PEGylated photosensitizer (PS) and OXA prodrug, which induced ICD and the phagocytosis of cancer cells via CD47 blockade. The combination of ICD induction and CD47 blockade improved DC maturation, T cell-mediated response, and antitumor immunity, which ultimately resulted in the regression of tumors, tumor metastasis, and prevention of tumor relapse. The inhibition of lymphatic metastasis in B16‐F10 model was attributed to combination therapy (**Figure 10**) 140. An obvious increase in DCs recruitment, tumor-infiltrating CTL (CD4+ and CD8+) as well as a significant decrease in T_reg_ cells was observed when immunotherapy combined with PDT. Therefore, more than 90% of tumor regression was attributed to iron NP-based alleviated immunosuppression and T cells infiltration 82.

TLR agonists and checkpoint blockade strategies are combined with photothermal and radiotherapy to achieve effective therapeutic efficacy and immunological memory 141, 142. The combination of NP-based sonodynamic therapy platform with checkpoint-blockade immunotherapy and immune adjuvant prevents tumor metastasis and induces antitumor and immune memory responses by provoking strong immune responses, including improved DCs maturation, infiltration of CD4+ and CD8+ lymphocytes, CD45+ leucocytes, and cytokine secretion 143. Therefore, the combination of NP-based immunotherapy with other therapeutic regimens may unleash the potential of cancer therapies. However, the immense attention should be paid on a deeper understanding of mechanisms underlying the immune systems and NPs-mediated toxicity.

## Conclusions and perspectives

Cancer immunotherapy has shifted the paradigm by delineating an alternative treatment regimen in order to thwart the development of metastasis. The studies have bolstered the concept that immune cells may play a dual role either heroes or villains in TME. On the other hand, there is circumstantial evidence that engineered NPs can promote the recruitment of immune cells in the tumor and overcome the anergy of tumor-specific T cells via blockade of immunosuppressive pathways. Besides modulating the immune response in TME, NP-based programming of immune cells improves the antitumor efficacy by enabling precise and persistent CTLs response. Programming of the immune system with engineered NPs has emerged as an effective strategy to improve antitumor immune response. TAMs contribute to promote tumor progression by taming protective adaptive immunity. The repolarization of TAMs in the tumor environment is, therefore, urgent to peruse. It is envisaged that the nanoparticle-mediated interplay between TME and immune system will significantly impact the clinical performance of cancer immunotherapy. However, a deeper understanding of mechanisms underlying the immune system and safety profiles of nanoparticles is required to avoid immune-related toxicity.

The non-persistent and autoimmune responses, lack of tumor-specific antigens and CTL, variation in checkpoint expression, and limited response to checkpoint blockade among patients are key challenges for cancer immunotherapy. The TAA-specific and persistence responses of CTL are crucial for inducing prolonged antitumor immunity. These limitations pose an urgent demand for combination strategies to simultaneously target immunostimulatory and immunosuppressive pathways to ensure effective therapeutic outcomes. The better understanding of the mechanisms underlying the tumor cells to evade immunity as well as to deleterious antitumor immune responses is required to defeat cancer. The acclaimed success of nanoparticle-based formulations in thwarting cancer is sometimes over exaggerated; however, we foresee a future in which defeat of tumor metastasis will be a mainstream practice.

A quiet large number of NP-based platforms are currently being evaluated in clinical trials for cancer immunotherapy (**Table 2**). A challenge in these platforms, however, is that most of the NP-based formulations are currently in phase 1 or 2. A range of approaches to modulate the immune system have been discussed in this article. These approaches present several opportunities for clinical translation. Notwithstanding, the animal models are mostly used to evaluate these strategies, making it challenging to translate the results from these models to humans. The choice of the humanized animal model may promote the translatability from preclinical studies to clinics. Choosing the correct nanocarriers is also crucial for nanomedicine-mediated cancer immunotherapy. For example, FDA-approved pharmaceutical excipients (e.g., lipid and specific polymers) are likely to get into the clinic faster than the unapproved materials. The biosafety and reproducibility of the nanovector formulation are also critical for the clinical translation of NPs-based cancer immunotherapy. When considering all of these issues, the survival rate in patients can be dramatically improved by broadening the spectra of cancer patients respond to immunotherapy.

## Figures and Tables

**Table 1:** Programming of the tumor microenvironment and immune system

NP platform	Immunotherapeuticagent	Function	Ref
**Programming of Tumor cells**			
Peptide assembling NPs	^D^PPA-1 NLG919	To block immune checkpoints and tryptophan metabolism	72
BCPN	OXA IDO inhibitor	To trigger ICD To relieve immunosuppression	75
Anti-CD47@CaCO_3_	Anti-CD47 antibody	To block the 'don't eat me' signal To activate the immune system TAM polarization	80
**Programming of APCs**			
sHDL nanodiscs	CSS-antigen (Cho-CpG)	To promote antigen presentation and induce DC maturation To elicit anti-tumor T-cell responses	83
iDR-NCs	CpG shRNA Neoantigen	To activate APCs To elicit neoantigen-specific T cells To induce antitumor immunity	90
**Programming of T cells**			
Polymer NPs	194-1BBz CAR Anti-CD3e f(ab′)2	To program tumor-specific circulating T cells Long-term tumor regression	105
Protein nanogels (NGs)	IL-15Sa Anti-CD45	To deliver TCR-signaling-responsive backpacks Selectively expanded T cells in tumors Substantial tumor growth inhibition	106
Lipid nanocapsules (NCs)	SN-38	To specifically target lymphoma cells To improve the therapeutic index of chemotherapeutics	109
**Programming of TAM**			
β-cyclodextrin NPs	R848	To achieve efficient TAM delivery To alter the myeloid phenotype To improve immune response	114
Iron oxide NPs (ferumoxytol)	Intrinsic therapeutic effect	To increase caspase-3 activity 'Off label' to protect from metastasis	115

**Table 2:** Nanoparticle-based cancer immunotherapy in clinical trials

Generic name	Nanoparticle platform	Cancer type	Status	Ref
JVRS-100	Lipid NP	Leukemia	Phase 1	144
DPX-Survivac	Liposome	Refractory diffuse large B-cell lymphoma	Phase 2	145
Lipovaxin-MM	Liposome	Metastatic melanoma	Phase 1	146
DPX-0907	Liposome	Advanced stage breast, ovarian and prostate cancer	Phase 1	147
WDVAX	PLGA	Metastatic melanoma	Phase 1	148
CYT-6091	Colloidal gold	Advanced solid tumors	Phase 1	149, 150
IMF-001	NP complex	Esophageal cancer	Phase 1	151, 152

## Abbreviations

ICD: immunogenic cell death
CRT: calreticulin
HMGB1: high-mobility group box 1 protein
ATP: adenosine triphosphate
HSP: heat-shock protein
DCs: dendritic cells
TLR: toll-like receptor
HSP: heat-shock protein
IL: interleukin
IFN: interferon
TAA: tumor-associated antigen
ROS: reactive oxygen species
MHC: major histocompatibility complex
NK: natural killer
NPs: nanoparticles
TAMs: tumor associated macrophages
TME: tumor microenvironment
FASL: FAS ligand
(TRAIL): (TNF)-related apoptosis inducing ligand
T_reg_ cells: regulatory T cells
MDSCs: myeloid-derived suppressor cells
TGFβ: transforming growth factor-β
IDO: indoleamine 2,3 dioxygenase
TNF: tumor necrosis factor
PD-L1: programmed death ligand-1
PD-1: programmed death protein-1
CTL: cytotoxic T-lymphocyte
CTLA-4: Cytotoxic T lymphocyte-associated antigen 4
MMP-2: matrix metalloproteinase-2
BCPN: binary cooperative prodrug nanoparticle
PDT: photodynamic therapy
OXA: oxaliplatin
PEG: polyethylene glycol
SIRPα: signal regulatory protein-α
APCs: antigen-presenting cells
HIF1-ɑ: hypoxia-inducible factor 1-ɑ
sHDL: high-density lipoprotein
Ag: antigen
CpG: cytosine-phosphate-guanosine oligonucleotides
cholesterol-modified CpG (Cho-CpG) PAMPs: pathogen-associated molecular patterns
PRR: pattern recognition receptors
CSS: cysteine-serine serine
EB: Evan blue
STAT3: signal transducer and activator of transcription3
shRNA: short hairpin RNA
MSRs: mesoporous silica rods
PEI: polyethyleneimine
OVA: ovalbumin
PLGA: pegylated poly(lactic-co-glycolic acid)
STING: stimulator of interferon genes
CAR T: chimeric antigen receptor therapy
NLS: nuclear localization signals
MTAS: microtubule-associated sequences
(TCR): T cell receptor
NGs: nanogels
IL-15Sa: interleukin 15 super-agonist
NCs: nanocapsules
NF-κB: nuclear factor-κB
ARG1: arginase-1
MRC1: mannose receptor-1
NOS2: nitric oxide synthase
CD: cyclodextrin
CLAN: cationic lipid-assisted nanoparticle
DAMPs: damage-associated molecular patterns
ER: endoplasmic reticulum
siRNA: small interfering RNA
PS: photosensitizer
